# Electrically-driven modulation of flow patterns in liquid crystal microfludics

**DOI:** 10.1038/s41598-024-53436-y

**Published:** 2024-02-28

**Authors:** Kamil Fedorowicz, Robert Prosser

**Affiliations:** School of Engineering, The University of Manchester, Manchester, M13 9PL UK

**Keywords:** Liquid crystals, Fluid dynamics, Computational methods

## Abstract

The flow of liquid crystals in the presence of electric fields is investigated as a possible means of flow control. The Beris-Edwards model is coupled to a free energy incorporating electric field effects. Simulations are conducted in straight channels and in junctions. Our findings reveal that local flow mediation can be achieved by the application of spatially varying electric fields. In rectangular straight channels, we report a two-stream velocity profile arising in response to the imposed electric field. Furthermore, we observe that the flow rate in each stream scales inversely with the Miesowicz viscosities, leading to the confinement of 70% of the throughput to one half of the channel. Similar flow partitioning is also demonstrated in channel junction geometries, where we show that using external fields provides a novel avenue for flow modulation in microfluidic circuits.

## Introduction

Microfluidics is a rapidly evolving interdisciplinary field that has revolutionised flow manipulation and analysis at the micro- and nanoscales^1^. Its applications include medical sensors^2^, drug delivery^3^, optofluidic modulators^4^, colour filters^5^, velocimetry^6^, and small-scale valves^7,8^. In contrast to traditional, bulky measurement devices, microfluidic instruments are more portable, offer more cost-effective analysis and can reduce the time required to conduct sample screening^9^.

Early microfluidic devices relied on Newtonian fluids^10^. The combination of small length scales and low characteristic speeds results in laminar flows at very small ($<<1$) Reynolds numbers^11,12^; in this regime, the stress is a linear function of the strain rate. This simple relation allows for the predictable and repeatable flow behaviour relied on by early chemical analysis devices^12^.

However, further developments of microfluidic devices have often relied on the exploitation of more complex *structured fluids* such as colloids and polymer solutions^13^. The micro-structural elements of these materials are large, and their response to deformation often involves both *strain* and *strain-rate* effects^14^. Structured fluids may therefore exhibit a number of rheological behaviours; in static configurations, normal stresses and memory effects may arise in response to microstructure deformation^14^; for flowing systems, the viscosity need not be constant and can depend on the shear rate^10,15^, surface treatment^16,17^ or external fields^18,19^. The interplay of these phenomena provides an opportunity to develop flow-control strategies, the success of which is predicated on a thorough understanding of the physics governing the fluids^10,20^.

In this work, we focus our attention on a sub-group of complex fluids, namely liquid crystals (LC). The anisotropic elements comprising the liquid crystal form structures with a directional order, whose mean orientation is called the *director*. Variable viscosity can be obtained by controlling director orientation through surface treatment^17^ or external fields^19^, and these have been used to drive the development of liquid crystal-based microfluidic devices^6,20,21^. Sengupta et al.^22^ were the first to control flow-director coupling via surface treatment, thereby regulating the velocity profiles in rectangular channels. Similar results have been obtained by Steffen et al.^23^ and by Fedorowicz and Prosser^24^, who have demonstrated the effects of microstructure elasticity on velocity profile and pressure drop.

In more complex domains, the competition between viscous and elastic effects, combined with geometric curvature, can be used to obtain a localised metering valve-like behaviour^25^. In extreme curvature cases (sharp right-angle bends), shear banding arises from the co-existence of subdomains where the director aligns either with the flow direction, or with the velocity gradient direction. The effective viscosity in the former region is much smaller and this produces a preferred path for most of the flow. A qualitatively similar effect was demonstrated by Sengupta et al.^22^ in straight channels, where shear bands were instead driven by temperature variations. The viscosity of liquid crystals is also strongly dependent on the presence of an electric field^19^. This observation was exploited by Na et al.^18^, who demonstrated electrically mediated local flow control in hierarchical branched channels; although their work was primarily experimental, simulation of the phenomenon was achieved using a Newtonian fluid with an inhomogeneous viscosity to mimic the effect of Miesowicz viscosities. Electrically programmed flows have also been used in the design of hierarchical filtration and sampling devices^26–28^.

The aim of this paper is to expand the functionality of electric fields in controlling microfluidic systems, and to build on recent related research^28–30^. We use homogeneous and non-homogeneous electric fields to control liquid crystal flows in rectangular channels as a potential means of flow control. Additionally, we consider LC flows through manifolds in the presence of non-homogeneous electric field in order to complement the experimental results of Na et al.^18^. Governing equations and geometries analysed in this paper are introduced in the “Methodology” section. The impact of the external fields on LC rheology and the flow modulation properties is discussed in the “Results” section. The paper summary and potential future developments are presented in the “Discussion” section.

## Methodology

### Governing equations

The fluid motion is described by the conservation equations of mass and linear momentum. The density of the liquid crystal fluid is assumed to be constant, and with incompressibility the continuity equation reduces to

1 $$\begin{aligned} {\varvec{\nabla }} \cdot {\textbf {u}} = {\textbf {0}} . \end{aligned}$$

${{\textbf {u}}}$ is the velocity vector, whose evolution is described through the linear momentum balance

2 $$\begin{aligned} {\rho } \frac{ D {{\textbf {u}}} }{ D {t} } = -{{\varvec{\nabla }}} {p} + {\varvec{\nabla }} \cdot \varvec{ \tau } , \end{aligned}$$

where ${\rho }$ is the density, $\frac{ D }{ D {t} }$ denotes the material derivative and *p* is the pressure. ${ \varvec{\tau } }$ represents the total (viscoelastic) stress tensor. In the case of liquid crystals, there is a coupling between the stress and microstructure arrangement; in the Beris-Edwards framework^31,32^, the stress tensor reads:

3 $$\begin{aligned} \varvec{ \tau }^{BE}= & {} { \mu } {\textbf { D }} - \xi \left[ \left( {\textbf {Q}} + \frac{ {\textbf {I}} }{3}\right) \cdot {\textbf {H}} + {\textbf {H}} \cdot \left( {\textbf {Q}} + \frac{ {\textbf {I}} }{3}\right) -2 \left( {\textbf {Q}} + \frac{ {\textbf {I}} }{3}\right) \left( {\textbf {H}}: {\textbf {Q}}\right) \right] \nonumber \\{} & {} + {\textbf {H}} \cdot {\textbf {Q}} - {\textbf {Q}} \cdot {\textbf {H}} + \varvec{ { \tau }}^{elast} + \varvec{ { \tau }}^{elec}. \end{aligned}$$

${\mu }$ is a Newtonian viscosity, ${\textbf {D}} = \frac{ {{\varvec{\nabla }}} {\textbf {u}} + ({{\varvec{\nabla }}} {\textbf {u}} )^T }{2}$ is the strain rate tensor and ${\textbf {H}}$ denotes the molecular field which arises in response to microstructure distortions. The order parameter tensor ${\textbf {Q}}$ describes the mean local orientation^16^; distortion in the orientational ordering is opposed by the elastic stress

4 $$\begin{aligned} { \tau }^{elast}_{\alpha \beta } =- \frac{\partial ^{2} { f}_{LdG} }{\partial {Q}_{ij, \alpha } \partial Q}_{ij,\beta } . \end{aligned}$$

${ f}_{LdG}$ is the Helmholtz free energy, whose exact form will be discussed later in the paper. Finally, the presence of an electric field produces an additional stress component dependent on the alignment between the external field and the order parameter tensor^28^

5 $$\begin{aligned} \varvec{\tau }^{elec}=\epsilon _0 ( \bar{\epsilon } {\textbf {E}}\otimes {\textbf {E}}+\epsilon _{mol} {\textbf {E}} \otimes ({\textbf {Q}}\cdot {\textbf {E}}) ). \end{aligned}$$

${\textbf {E}}$ denotes the electric field, $\epsilon _0$ and $\bar{\epsilon }$ are the vacuum and the average nematic permittivity, and $\epsilon _{mol}$ is the molecular anisotropy^33^.

Computation of the order parameter tensor ${\textbf {Q}}$ is achieved via an angular momentum equation balancing viscous torques against the molecular field^31,34^:

6 $$\begin{aligned} \frac{D {\textbf {Q}} }{D {t} }= {\textbf {S}} + {\Gamma } {\textbf {H}} . \end{aligned}$$

The viscous torque acting to rotate the nematic element towards the Leslie angle^34,35^ (controlled by the tumbling parameter $\xi$) is given by

7 $$\begin{aligned} {\textbf {S}} = ( \xi {\textbf {D}} - \varvec{ {\omega } } ) \cdot \left( {\textbf {Q}} + \frac{ {\textbf { I }} }{3} \right) + \left( {\textbf {Q}} + \frac{ {\textbf { I }} }{3} \right) \cdot \left( \xi {\textbf {D}} + \varvec{ {\omega } } \right) - 2\xi \left( {\textbf {Q}} + \frac{ {\textbf { I }} }{3} \right) {\textrm{tr}}\left( {\textbf {Q}} \cdot {{\varvec{\nabla }}} \varvec{{u}} \right) , \end{aligned}$$

where ${\varvec{\omega }} = \frac{ {{\varvec{\nabla }}} {\textbf {u}} - ({{\varvec{\nabla }}} {\textbf {u}} )^T }{2}$ is the vorticity. $\Gamma$ is the rotational viscosity and the competing molecular field ${\textbf {H}}$ is defined as^31,35^

8 $$\begin{aligned} {\textbf {H}}= -\frac{\delta { f}_{LdG} }{\delta {\textbf {Q}}} + \frac{1}{3} {\textrm{tr}}\frac{\delta { f}_{LdG} }{\delta {\textbf {Q}} } {\textbf {I}} , \end{aligned}$$

where $\frac{\delta }{\delta {\textbf {Q}}}$ denotes the functional derivative. In the absence of flow effects, the action of ${\textbf {H}}$ minimises the Helmholtz free energy, denoted by ${f}_{LdG}$, which in the Beris-Edwards framework has distortional, bulk-free nematic and electric components:

9 $$\begin{aligned} { f}_{LdG}= & {} { f}^Q_{elastic} +{ f}_{nematic} + { f}_{electric} = \frac{1}{2} {K} {Q}_{ik,j} {Q}_{ik,j} + \frac{ {a} }{2} {\textrm{tr}}({\textbf {Q}}\cdot {\textbf {Q}} ) - \frac{ {b} }{3} {\textrm{tr}}( {\textbf {Q}} \cdot {\textbf {Q}} \cdot {\textbf {Q}} ) \nonumber \\{} & {} + \frac{ {c} }{4} {\textrm{tr}}^2({\textbf {Q}} \cdot {\textbf {Q}} ) - \frac{1}{2} \epsilon _0 \left( \bar{\epsilon } {\textbf {E}} \cdot {{\textbf {E}}} + \epsilon _{mol} {\textbf {Q}}: ({{\textbf {E}}} \otimes {{\textbf {E}}}) \right) . \end{aligned}$$

${K}^Q$ is an elastic constant and ${a},\ {b}, \ {c}$ are phenomenological parameters^16,36^. The director orientation is governed by the competition between elastic and flow effects, the relative strength of which is expressed through the Ericksen number: $Er = \frac{ {u}_0 {L}_0 }{ {\Gamma } {K} }$, where ${u}_0$ and ${L}_0$ are the velocity- and length-scales relevant to the problem. In our simulations we use ${u}_0$ as the mean flow speed in the channel, while ${L}_0$ is the channel height.

In the absence of orientational distortions and electric fields, the ordering of a static nematic sample is governed by the bulk free energy. The molecular field contribution of the bulk free energy has no impact on the preferred orientation^24^, and its action is limited to driving the system towards the order parameter that minimises ${ f}_{nematic}$, given by

10 $$\begin{aligned} Q_{eq} = \frac{ {b} + \sqrt{ {b}^2 - 24 {a} {c} } }{ 4 {c} }. \end{aligned}$$

The competition between the flow and nematic effects acting on the order parameter is expressed through the Deborah number $De = \frac{ {u}_0 }{ {\Gamma } b {L}_0 }$. Since ${b} \approx 10^6 \text { J/m}^3$, $u_0\approx 10^{-4} \text { m/s}$ and $L_0 \approx 10^{-4} \text { m}$ in typical LC flows^22^, this paper considers only $De<<1$ flows, as this regime is relevant to microfluidic applications.

At small Deborah numbers, the bulk free energy drives the system towards the uniaxial state, in which the order parameter tensor can be represented as^33^

11 $$\begin{aligned} {\textbf {Q}} = Q_{eq} \left( {\textbf {nn}} - \frac{ {\textbf {I}} }{ 3 } \right) , \end{aligned}$$

where ${\textbf {n}}$ is the *director* and represents the local mean orientation of the nematic elements.

The last term in Eq. (9) represents the contribution of the electric field to the Helmholtz free energy. Since the term $\bar{\epsilon } {{\textbf {E}}} \cdot {{\textbf {E}}}$ is independent of ${\textbf {Q}}$, it does not impact ordering and is therefore often omitted^17^. The nature of electric-nematic interactions is qualitatively described by the sign of $\epsilon _{mol}$; positive (negative) $\epsilon _{mol}$ drives the nematic axes to align parallel (perpendicular) to the direction of the external field^17^. In this paper, the relative importance of viscous to electric effects is measured by the Hartman number $Ha = \frac{ {u}_0 }{ {\Gamma } {L}_0 \epsilon _0 \epsilon _m ({\textbf {E}} \cdot {\textbf {E}}) }$.

Due to the anisotropic properties of liquid crystals, there is a two-way coupling between their alignment and the electric field^37,38^, which increases the complexity of the problem. In order to simplify the analysis, we follow the approach taken in previous works concerned with the electro-rheological properties of liquid crystals^28,39–41^, and assume that the electric field is independent of the director orientation. This simplification was found to have little effect on the director arrangements in static problems that involve the competition between electric and elastic effects^17,42^.

### Numerical solution

Numerical solutions were obtained using the OpenFOAM solver *rheoFoamLC*^43^. The solver has been previously used to model liquid crystal flows in other complex geometries^44^, and is capable of capturing defects^25,45^. Combination of the extended definition of the free energy (Eq. (9)) with Eq. (8) provides a modification of the angular momentum equation (Eq. (6)) that incorporates the electric field.

####  Geometries

**Figure 1:**
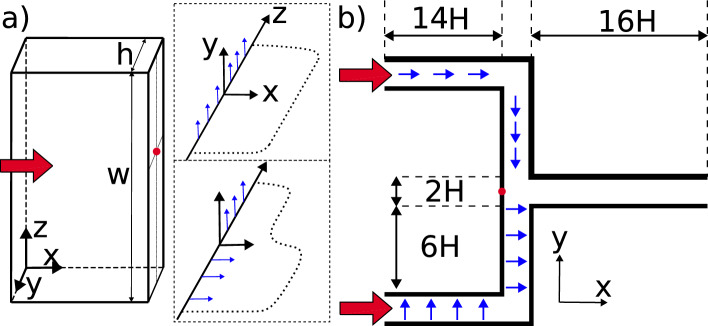
Geometries considered in this paper: (**a**) rectangular channel; (**b**) two-dimensional manifold with two inlet channels. Blue arrows indicate the direction of the applied electric field and red arrows denote inlet locations. The origin of the coordinate system is denoted with red dot. High aspect ratio rectangular channels with $w=10h$ are considered and we have set $h=H=10 \mu \text {m}$ throughout all simulations. Insets in (**a**) illustrate the direction of applied electric field and the expected velocity profiles (shown dotted); a further discussion will be presented in the “Results” section.

We consider the flow of liquid crystals in the following geometries:

1. Fully developed three dimensional channels depicted in Fig. 1a (the reference frame, test configurations and relevant nomenclature used throughout the paper is also displayed). Due to the high aspect ratio ($w/h=10$), the velocity gradient (and thus the shear stress) in the *y* direction is much larger than its *z* counterpart. For this reason, we impose a uniform $y-$ aligned electric field of varying strength (upper inset in Fig. 1a), as this direction is able to produce a wide spectrum of effective viscosities; a schematic of the typical velocity profile is also provided in the inset. Additionally, we consider flows with a non-uniform electric field, as depicted in the lower inset of Fig. 1a. By introducing a flow-aligned electric field in the bottom half of the channel, we expect locally to promote the flow—the schematic velocity profile here reflects this expectation.
2. Simple manifolds. Studies of this geometry are inspired by the previous work of Na et al.^18^, whose experiments on manifolds with four branches demonstrate the control of the local flow rate via the electric field. Here, we consider a simplified design, with a manifold consisting of two inlet channels feeding into an outlet channel. We explore flow programming capabilities by varying the alignment between the electric field, the flow field and the nematic axes. The electric field is aligned in the flow direction in the upper channel, and in the velocity gradient direction in the lower channel (depicted by the arrows in Fig. 1b). The strength of the field in each limb is also a control variable.

*Boundary and initial conditions* We use no-slip boundary conditions for the velocity on all walls in each configuration. Perfect ordering of the LC is assumed on the boundary $(Q=1)$ and homeotropic anchoring with infinite strength is imposed in the rectangular channel; this is consistent with our previous research^24,25,44,45^ and other researchers who have investigated the rheology of liquid crystals (Denniston and Yeomans^32,34^, Rey and coworkers^46–48^, and Sengupta and coworkers^22,23^).

A fixed pressure gradient is imposed in the straight channel simulations to drive the flow. The velocity scale is calculated based on $\frac{\partial p}{\partial x}$ as $\tilde{u}_0^{channel} = \frac{\partial p}{\partial x} \frac{h^2}{\Gamma }$. In the case of connecting junctions simulations, a fixed and equal pressure is imposed at both inlets, and a zero pressure BC is imposed at the outlet. The selection of the fixed pressure boundary condition reflects the action of real pumps. In order to ensure consistent comparisons between flows driven by the identical pressure differences, we define the characteristic velocity scale as $\tilde{u}_0^{junction} = \frac{ \Delta p }{ 34 {H} } \frac{H^2}{\Gamma } = \frac{ \Delta p }{ 34 } \frac{H}{\Gamma }$, where $\frac{ \Delta p }{ 34 {H} }$ is the average pressure gradient across the whole geometry and $34H(=24H \text { horizontal}+6H \text { vertical})$ provides a scale for the centre-line channel length across which the pressure drop $\Delta p$ takes place. Zero gradient velocity boundary conditions are imposed at both inlet and outlet.

All simulations are initialised with the ${\textbf {Q}}-$tensor in the isotropic state and a zero velocity field. We expect the initial condition to have little impact on the steady state behaviour because of the relatively high Ericksen number flows considered in this work.

*Computational domain* The fully developed three dimensional channel flow is calculated using a two-dimensional rectangular structured mesh $\Delta y =40^{-1}h$, $\Delta z =400^{-1}w=20^{-1}h$, where $\Delta y$ and $\Delta z$ are the length scales of the volume element in the $y-$ and $z-$ directions, respectively. Finally, the same mesh density was used in the simulations of the connecting junctions; $\Delta y=\Delta x=40^{-1}H$. Grid independent solutions have been obtained throughout.

*Material parameters* Material parameters used in all simulations are of similar values to those reported in previous studies^22,25^: $\mu =0.2 \ \text {Pa}\cdot \text {s}$, $K=40 \ \text {pN}$, $a=-0.2 \ \text {MJ}/\text {m}^3$, $b=4 \ \text {MJ}/\text {m}^3$, $c=4 \ \text {MJ}/\text {m}^3$, $\xi =1$, $\Gamma =7 \ (\text {Pa}\cdot \text {s})^{-1}$; these values are representative of the 5CB liquid crystal, which is frequently used in microfluidic research^49,50^.

For the given set of the bulk free energy constants $a, \ b, \ c$, the calculation of the equilibrium order parameter with Eq. (10) gives $Q_{eq}=0.62$. A mapping between the Beris-Edwards and Leslie-Ericksen models (see refs^24,32^ for details) enables calculation of the Miesowicz viscosities, which are $0.21 \ \text {Pa}\cdot \text {s}$ and $0.52 \ \text {Pa}\cdot \text {s}$ for the flow and velocity-gradient aligned uniform orientations, respectively. These values will become relevant in later sections to estimate the degree of flow imbalance in cases where the non-uniform director fields are encountered.

## Results

### Impact of the flow and electric fields on the director alignment

Following a similar procedure as in our previous work^24^, we can separate the effects contributing to the evolution of the microstructure orientation and to the order parameter. In the uniaxial, constant order parameter limit, the time derivative of the ${\textbf {Q}}$ tensor evolution depends only on the director evolution: $\frac{D {\textbf {Q}} }{Dt} = Q \frac{D {\textbf {nn}} }{Dt}$. Assuming $Q=1$, and representing both the director and electric field vectors in terms of their individual polar angles with respect to the flow: (${\textbf {n}} = [\cos (\theta ), \ \sin (\theta ), 0]$), ${\textbf {E}}=|{\textbf {E}}| [\cos (\alpha ), \ \sin (\alpha ),0]$), $\frac{D {\textbf {Q}} }{Dt}$ can be expressed as:

12 $$\begin{aligned} \frac{D {\textbf {Q}} }{Dt} = \left[ \begin{array}{ccc} - \sin (2\theta ) &{} \cos (2\theta )&{} 0 \\ \cos (2\theta ) &{} \sin (2\theta ) &{} 0 \\ 0 &{} 0 &{} 0 \end{array}\right] \frac{ D \theta }{ D t} = {\textbf {B}} \frac{ D \theta }{ D t} . \end{aligned}$$

Noting that ${\textbf {B}}:{\textbf {B}}=1$, we can obtain the evolution equation for the director angle by taking the double contraction of ${\textbf {B}}$ with each contribution of the angular momentum equation (Eq. 6). The contribution of the electric field to the angular momentum balance is given by

13 $$\begin{aligned} H_{electric} = \Gamma {\textbf {H}} (f_{electric}) : {\textbf {B}} =\Gamma \epsilon _0 \epsilon _m |{\textbf {E}}|^2 \sin (2(\alpha -\theta )) . \end{aligned}$$

$\alpha -\theta$ measures the misalignment between ${\textbf {E}}$ and ${\textbf {n}}$. Similarly, the contribution of viscous effects to the angular momentum balance in a one-dimensional flow reads

14 $$\begin{aligned} S = {\textbf {S}} : {\textbf {B}} = -2 \frac{\partial u}{\partial y} \sin ^2 \theta . \end{aligned}$$

The contribution of viscous effects vanishes when the director aligns in the flow direction ($\theta =0$). The impact of elastic effects is given by

15 $$\begin{aligned} H_{elastic} = \Gamma {\textbf {H}} (f_{elastic}) : {\textbf {B}} =\Gamma K \nabla ^2 \theta . \end{aligned}$$

It is worth noting that since ${\textbf {H}} (f_{nematic}): {\textbf {B}} = 0$, the nematic energy contribution has no impact on the director orientation. Combining Eqs. (12–15), we construct a transport equation for the director angle, which in dimensionless form reads

16 $$\begin{aligned} \frac{\partial \theta }{\partial t} = Ha^{-1} \sin (2(\alpha -\theta )) -2 \frac{\partial u}{\partial y} \sin ^2 \theta + Er^{-1} \nabla ^2 \theta . \end{aligned}$$

In general, the director is oriented at the free-stream angle $\theta _{\infty }$ far from the wall, while the boundaries impose homeotropic alignment. For a strong $(Ha<<1)$ electric field, the two zones are separated by a well defined boundary layer. Since the electric field dominates the flow contribution ($|Ha^{-1} \sin (2(\alpha -\theta ))|>>|2 \frac{\partial u}{\partial y} \sin ^2 \theta |$), director distortions arise solely from the misalignment between ${\textbf {E}}$ and ${\textbf {n}}_{wall}$ (for $\alpha \ne \frac{\pi }{2}$). The boundary layer thickness can be then estimated from Eq. (16) as $\delta \approx O(\sqrt{\frac{Ha}{Er} })=O(\sqrt{\frac{ K }{ L_0^2 \epsilon _0 \epsilon _m ({\textbf {E}} \cdot {\textbf {E}} ) } })$; this result has been confirmed by numerical simulations (not reported here).

**Figure 2:**
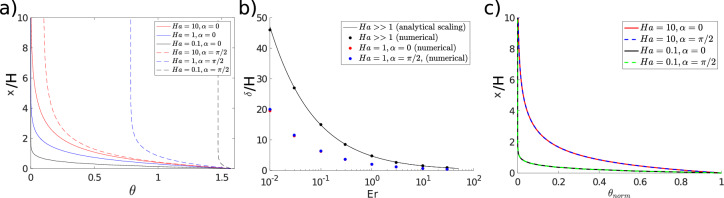
(**a**) Spatial variation of the director angle for different *Ha* and $\alpha$ at $Er=1$; (**b**) boundary layer thickness ($\delta$ is measured as the layer width where 95% variation of the director angle occurs) as a function of *Er*; (**c**) normalised director angle for different *Ha* and $\alpha$ at $Er=1$. $\frac{\partial u}{\partial y}=1$ throughout all simulations for simplicity.

Sufficiently far from the wall, the director angle satisfies $Ha^{-1} \sin (2(\alpha -\theta )) -2 \frac{\partial u}{\partial y} \sin ^2 \theta =0$, so $\theta$ depends on the direction and strength of the electric field. When ${\textbf {E}}$ is aligned with the flow ($\alpha =0$), $\theta _{\infty } =0$ irrespective of the Hartman number, as shown by the continuous lines in Fig. 2a. Conversely, when ${\textbf {E}} || {\textbf {n}}_{wall}$ ($\alpha =\pi /2$), the free stream angle is a function of the Hartman number

17 $$\begin{aligned} \theta _{\infty } = \tan ^{-1}(Ha^{-1}) . \end{aligned}$$

The result indicates that as the electric field becomes stronger (*Ha* decreases), the director is driven towards the wall-imposed alignment (dashed lines Fig. 2a) because the combination of electric field and homeotropic anchoring overcomes viscous torques. The boundary layer disappears in the limit $Ha^{-1} \rightarrow \infty$ (*Ha* effects $>>$ strain rate effects), where the director is aligned in the wall-normal direction throughout the domain.

When neither the flow nor the electric contributions are negligible, no clear limiting behaviour can be established and Eq. (16) must be solved numerically. Figure 2b shows that similarly to flows where ${\textbf {E}}={\textbf {0}}$, the boundary layer becomes thinner as the Ericksen number increases. The presence of electric fields further strengthens the Ericksen number effect. The collocation of red and blue dots in Fig. 2b show that $\delta$ reduces both when ${\textbf {E}} || {\textbf {u}}$ and ${\textbf {E}} || {\textbf {n}}_{wall}$, indicating that the direction of ${\textbf {E}}$ has little impact on $\delta$. The boundary layer is largest when the electric fields disappear ($Ha \rightarrow \infty$), and $\delta \propto Er^{-0.5}$ as described by the de-Gennes shear flow characteristic length^16,22^. Re-scaling the wall normal direction by $\tilde{y} = y\sqrt{Er}$ removes the explicit dependency of Eq. (16) on the Ericksen number; in the re-scaled coordinates the boundary layer structure depends only on the (modified) strain rate and Hartman number.

Depending on the orientation of ${\textbf {E}}$, there are different mechanisms that drive the reduction in the boundary layer thickness. When ${\textbf {E}}$ is aligned with the flow, $\delta$ decreases because the electric effects strengthen the viscous contribution, so the orientational transition of ${\textbf {n}}$ occurs over a narrower region. Conversely, when ${\textbf {E}} || {\textbf {n}}_{wall}$, $\delta$ decreases because the director rotates over a smaller distance (from $\pi /2$ to $\tan ^{-1} (Ha)$) to reach the free-stream value. Figure 2c shows that when the director angle is appropriately scaled $\theta _{norm} = \frac{\theta - \theta _\infty }{\theta _{wall} - \theta _\infty }$, director profiles collapse onto each other, which shows that the boundary layer scaling is independent of the direction of the electric field.

Analysis conducted in this paper uses $\xi =1$, in which limit the Beris-Edwards model produces a fixed steady state solution in the absence of electric fields^34^. This need not be the case when $\xi <1$, where the Beris-Edwards model can produce director tumbling, or metastable states oriented in the vorticity direction^51,52^. In the Supplementary Information section, we show that the action of the electric field interferes with these effects by affecting the oscillatory behaviour (Figs. S1 and S2 in the SI section). In the limit of a very small Deborah number (in which case tumbling effects are the strongest), the critical Hartman number to promote the oscillatory behaviour scales with the inverse of the equilibrium order parameter. Otherwise, when the ordering effects are weak ($De=O(1)$), there are periodic variations in both the director angle and the order parameter that are damped by the electric field (Figs. S3 and S4 in the SI). Figure S5 in the SI shows that the critical Hartman number to promote a fixed value solution only weakly depends on the Deborah number.

### Rectangular channel with a uniform electric field

**Figure 3:**
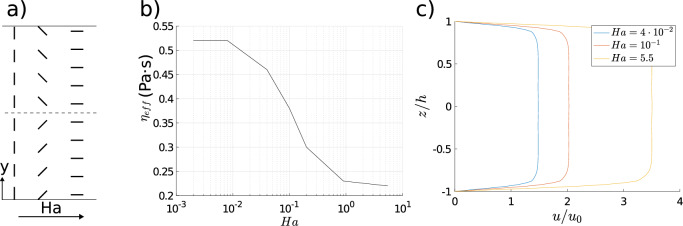
(**a**) Schematic depiction of director arrangement depending on the Hartman number (increasing *Ha* to the right); (**b**) effective viscosity ($\text {Pa} \cdot \text {s}$) as a function of the Hartman number; (**c**) Velocity profile at $y=0$ for a range of Hartman numbers. $Er=100$ throughout all simulations.

For the high aspect ratio channels ($w>>h$) considered in this paper, there are much larger velocity gradients in the $y-$direction than the $z-$ direction. Therefore, for a fixed pressure gradient, the flow speed is more sensitive to changes in the effective viscosity in the *xy* plane. Imposing a $y-$aligned electric field provides a wide spectrum of director orientations depending on *Ha* (Fig. 3a). The resultant effective viscosities range between minimum and maximum Miesowicz viscosities ($0.21 \text {Pa} \cdot \text {s}$ and $0.52 \text {Pa} \cdot \text {s}$, respectively), as shown in Fig. 3b. The viscous resistance is reflected in the velocity profile, which for a fixed pressure gradient, can have different maxima depending on the strength of effective field (Fig. 3c). Velocity profiles have similar shapes due to the high Ericksen number limit, in which elasticity has little impact on the director arrangement and the flow; this need not be the case when $Er=O(1)$. Finally, our result confirms that the velocity gradient is small in the *xz* plane with the exception of the near-wall boundary layer. As a result, the majority of the viscous resistance within the channel arises in response to shear in the *xy* plane; this confirms that an application of the $y-$aligned electric fields is optimum for flow programming.

### Rectangular channel with a non-uniform electric field

**Figure 4:**
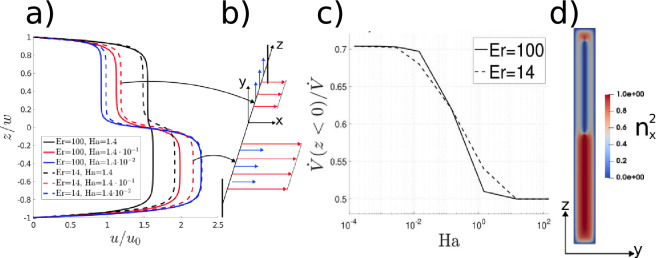
(**a**) Effect of the applied external field (glyphs) on the centreline velocity profile as a function of Hartman and Ericksen numbers; (**b**) schematic illustration of the effect of electric field (blue arrows) on the velocity (red arrows) at small *Ha*; (**c**) throughput fraction ($\dot{V}$) through the lower half of the channel at $Er>>1$ and $Er=14$; d) flow alignment (measured by $n_x^2 = \cos ^2(\theta )$) at $Ha=1.4 \cdot 10^{-1}$ at $Er=14$.

In this section, a nematic’s sensitivity to an imposed electric field is coupled the induced anisotropic viscosity to generate spatially non-uniform viscosity distributions. ure 4a provides velocity predictions for cases where the electric field variation occurs in the spanwise (z) direction. At $Ha<<1$ and $Er \sim O(10)$, the electric field dominates flow driven and elastic effects. Figure 4b shows that for $z/w>0$, the director aligns with the velocity gradient direction; for $z/w<0$, alignment is in the flow direction. The resulting viscous inhomogeneity leads to the two-stream velocity profile denoted by the red and blue lines in Fig. 4a; the peak flow speed occurs in the lower half of the domain where ${\textbf {u}} || {\textbf {E}}$. As the Hartman number increases, the flow contribution becomes dominant and the director remains nearly aligned with the flow throughout the channel; the effective viscosity across the channel is nearly uniform, and so is the throughput (black continuous line in Fig. 4a).

An application of the electric field can be used locally to promote the flow in selected locations: at small *Ha*, over 70% of the throughput occurs in the lower half-channel, as shown in Fig. 4c. This value is consistent with a simple estimate based on the assumption that the throughput is inversely proportional to the local viscosity. For the material parameters selected in this paper, we expect 71%($=\frac{0.52}{0.52+0.21}$) of the throughput to occur in the channel’s lower half, where the director aligns in the flow direction (Fig. 4d). Equal throughput distribution is recovered at high *Ha*; switching between equal and unequal flow rates in each channel halves occurs here at around $Ha \sim O(10^{-1})$. As the Ericksen number increases, the impact of the elasticity can be neglected, so the switchover between the equal and unequal flow distributions becomes quicker; this is confirmed in Fig. 4c (continuous line line).

### Manifolds

A natural application of the observed flow asymmetries is as a type of metering valve. As a prototype we apply an inhomogeneous electric field to a manifold configuration, with the field oriented in the velocity (velocity gradient) directions in the upper (lower) channels, respectively. In practice, a spatially variable electric fields on small scales can be generated through dielectrophoresis microfludic chips^53^. A constant pressure difference is applied between the inlet and outlet channels, and we only modify the strength of the electric field. A zero gradient boundary condition for the ${\textbf {Q}}-$tensor is imposed on all boundaries so that the analysis can focus on the competition between viscous and electric effects^16^.

**Figure 5:**
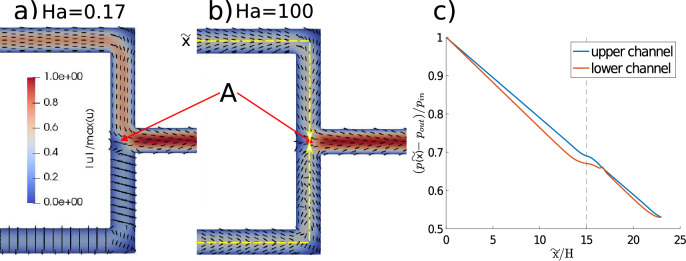
Velocity contour plot and glyphs of the director field at (**a**) $Ha = 0.17$; (**b**) $Ha>>1$; (**c**) relative pressure drop in the upper and lower channels between the inlet and the entrance to the outlet channel (denoted by the letter A the contour plots) at $Ha=0.17$; the vertical line denotes the bend location. $\tilde{x}$ is the distance from the entrance along the channel centreline, and is schematically illustrated by the yellow line in (**b**).

Figures 5a and 5b demonstrate that the distribution of incoming streams can be mediated by the imposed electric fields. At low *Ha*, significant discrepancies in throughput between the two inlet channels are observed; up to 70% of the total flow occurs in the flow-aligned branch. The $\dot{V}(z>0)/\dot{V}$ vs *Ha* dependency varies in the same manner as in the case of straight channels (Fig. 4c), and hence the plot is not included. This similarity suggests that physical barriers (such as walls) are not necessary in order to obtain flow partitioning provided that the electric field is sufficiently strong.

Figure 5c shows the pressure variations at the centreline between the inlet and the point where the manifold branches meet (denoted by the letter A in Fig. 5a, b). Despite the pressure drop in the upper and lower branch is identical, it appears that the pressure drop around the bend region (around $x/H=15$) is much smaller in the lower channel at low *Ha*. This is due to the flow-aligned director field in the corner of the lower channel (Fig. 5a), which in combination with small flow speeds (lower than in the analogous location in the upper branch) produces a small pressure drop around the corner. The result suggests that there can be differing patterns in the pressure drop depending on the direction of the electric field, and a detailed investigation of this phenomenon is a subject of the future work.

## Discussion

In this study we have investigated the physics of liquid crystal flow modulation with the use of electric fields. In flows with homeotropic anchoring, a uniform electric field aligned with the velocity amplifies Ericksen number effects, resulting in a narrower boundary layer where the director reorients. In contrast, elastic effects are strengthened when the electric field is parallel to the wall anchoring, and the director aligns at an *Ha*-dependent angle far away from the boundary. Similarly to the ${\textbf {E}} || {\textbf {u}}$ case, the director boundary layer shrinks, which happens because the director rotates over a smaller angular distance. The mechanism of externally-controlled director alignment can be used for to manipulate the effective viscosity and thus the flow rate in rectangular channels.

The situation becomes more complex when the external field is non-uniform; both viscous and elastic effects may be relatively influenced, and the ultimate outcome is closely linked to the exact configuration of the field. With appropriate control, an uneven throughput distribution within the channel is produced, leading to a type of electrically-driven shear banding; this novel phenomenon has not, to the best of our knowledge, been previously demonstrated in liquid crystal flows. Shear banding manifests as a distinct two-stream velocity profile in straight channels, with the velocity in each partition being inversely proportional to the local viscosity. In typical liquid crystals, $\eta _{\perp }$ can exceed $\eta _{||}$ by a factor of five^17^, providing significant potential for flow tuning.

Finally, we have demonstrated how the electric fields can be used in simple manifolds as a type of metering valve in order to control the flow proportions in mixing applications such as drug delivery. It remains an open question whether the concepts presented in this work may be extended to more complex geometries (e.g. manifolds consisting of more than two branches). Such configurations would improve the ability to control mixing operations of multiple fluid streams at a fixed pumping load.

We identify two primary directions for the future work: (1) investigations of manifolds and similar geometries in three dimensions, while accounting for the elastic effects at the wall; (2) incorporation of the Maxwell equations to the problem in order to obtain more accurate solutions. The latter point will be particularly important in situations where the electric field varies spatially.

### Supplementary Information

Supplementary Information.

## Data Availability

All data generated or analysed during this study are included in this published article and the Supplementary Information.
