# Supplementary material for: Electrically-driven modulation of flow patterns in liquid crystal microfludics
Source: Sci Rep. 2024 Feb 28;14:4875. doi: 10.1038/s41598-024-53436-y (PMC10901866; doi:10.1038/s41598-024-53436-y)
Supplement: Supplementary file 1 — Supplementary Information. [file 41598_2024_53436_MOESM1_ESM.pdf]

Supplementary information to the paper 'Electrically-driven modulation of flow patterns in liquid crystal microfludics'

Kamil Fedorowicz[1,*+] **and Robert Prosser**[1+]

[1]School of Engineering, The University of Manchester, Manchester M13 9PL, UK.
[*]kamil.fedorowicz@manchester.ac.uk
[+]these authors contributed equally to this work

1 Microstructure evolution

The analysis presented below assumes that the liquid crystal remains in the uniaxial state, but we allow for a variable order parameter Q. We consider a one-dimensional shear flow, similar to other studies[1]; the order parameter tensor is given by

$$\mathbf{Q} = Q(\mathbf{nn} - \mathbf{I}/3), \tag{1}$$

where $\mathbf{n} = [\cos(\theta),\ \sin(\theta), 0]$, and Q is a variable order parameter. The time derivative of $\mathbf{Q}$ can be decomposed into the $Q-$ and $\theta-$dependent components:

$$\frac{D\mathbf{Q}}{Dt} = (\mathbf{nn} - \mathbf{I}/3)\frac{DQ}{Dt} + Q\begin{bmatrix} -\sin(2\theta) & \cos(2\theta) & 0 \\ \cos(2\theta) & \sin(2\theta) & 0 \\ 0 & 0 & 0 \end{bmatrix}\frac{D\theta}{Dt} = \mathbf{A}\frac{DQ}{Dt} + \mathbf{B}\frac{D\theta}{Dt}. \tag{2}$$

Since $\mathbf{A} : \mathbf{B} = 0$, we can obtain the evolution equations for the order parameter and the director angle by taking the double contraction of eq. (2) with either $\mathbf{A}$ or $\mathbf{B}$, respectively:

$$\frac{\partial Q}{\partial t} = \frac{2Q}{3Ha}\left[2 + 3\cos 2\theta \cos^2\alpha + \frac{3}{2}\sin 2\alpha \sin 2\theta - 3\cos^2\theta\right] - [\frac{4c}{9b}Q^4 - \frac{2}{9}Q^3 + \frac{2a}{3b}Q^2]De^{-1} - \frac{\sin 2\theta(2Q^2 - Q - 1)Q\xi\dot{\gamma}}{3}, \tag{3}$$

$$\frac{\partial \theta}{\partial t} = \sin(2(\alpha - \theta))Ha^{-1} + \frac{2\dot{\gamma}}{3}\left(\xi \cos 2\theta \left\{1 + \frac{Q}{2}\right\} - \frac{3Q}{2}\right), \tag{4}$$

where $a,\ b,\ c$ are the parameters of the nematic energy, Ha is the Hartman number, and $\dot{\gamma}$ is the shear rate. Note that in the limit of $Q = 1$, $\xi = 1$, eq. (4) reduces to eq. (16) of the main manuscript with the elastic effects neglected.

1.1 $De << 1$ limit

The order parameter is constant (and equal to Q_{eq}) in the $De << 1$ limit, and the behaviour of the system is solely described by the evolution equation for the director angle:

$$\frac{\partial \theta}{\partial t} = \sin(2(\alpha - \theta))Ha^{-1} + \frac{2\dot{\gamma}}{3}\left(\xi \cos 2\theta \left\{1 + \frac{Q_{eq}}{2}\right\} - \frac{3Q_{eq}}{2}\right). \tag{5}$$

The qualitative response of the system strongly depends on the tumbling parameter ξ. When $\xi \geq 1$, the director reaches a fixed angular orientation in the absence of an external field[2]. When the electric field is present, its effect on the resultant director angle depends its strength relative to the viscous effects. When $\xi < 1$, oscillations may occur; their character typically depends on the Ha regime.

1.1.1 $\xi = 0$
In the limit $\xi = 0$, the system is most susceptible to tumbling. For this case, we can simplify further the θ evolution equation to

$$\frac{\partial\theta}{\partial t} = \sin(2(\alpha - \theta))Ha^{-1} - \dot{\gamma}Q_{eq}. \tag{6}$$

A steady state solution is achieved if

$$\theta = \alpha - \frac{1}{2}\sin^{-1}(Q_{eq}\dot{\gamma}Ha). \tag{7}$$

Eq.(7) shows the role of the electric field in enforcing a stationary solution; increasing the field strength ($Ha \to 0$) drives the director towards perfect co-alignment. Conversely, when the electric field is sufficiently weak ($Ha > Q_{eq}\dot{\gamma}$), no such steady state can be achieved.

1.1.2 $\xi \neq 0$
For this case, the qualitative behaviour of the system also depends on the alignment between the electric field and the deformation axes.

Assuming that the electric field is oriented in the flow direction ($\alpha = 0$), the θ evolution equation becomes

$$\frac{\partial\theta}{\partial t} = -\sin(2\theta)Ha^{-1} + \frac{2\dot{\gamma}}{3}\left(\xi\cos 2\theta\left\{1 + \frac{Q_{eq}}{2}\right\} - \frac{3Q_{eq}}{2}\right). \tag{8}$$

The solution to this equation for the steady state is

$$\sin(2\theta) = -\frac{\xi(Q+2)\sqrt{9 + (\xi^2(Q+2)^2 - 9Q^2)Ha^2} + 9Q}{9 + Ha^2(Q+2)^2\xi^2\dot{\gamma}^2}\dot{\gamma}Ha. \tag{9}$$

When the external field is sufficiently strong, fixed steady state solutions may be obtained for a range of values of (Q, ξ). As the field strength weakens ($Ha >> 1$), θ becomes complex and the solution becomes oscillatory; the oscillation frequency increases with increasing Hartmann number. Representative plots of the stationary/oscillatory regions in the $Ha - Q$ space are illustrated in fig. S1(a-c).

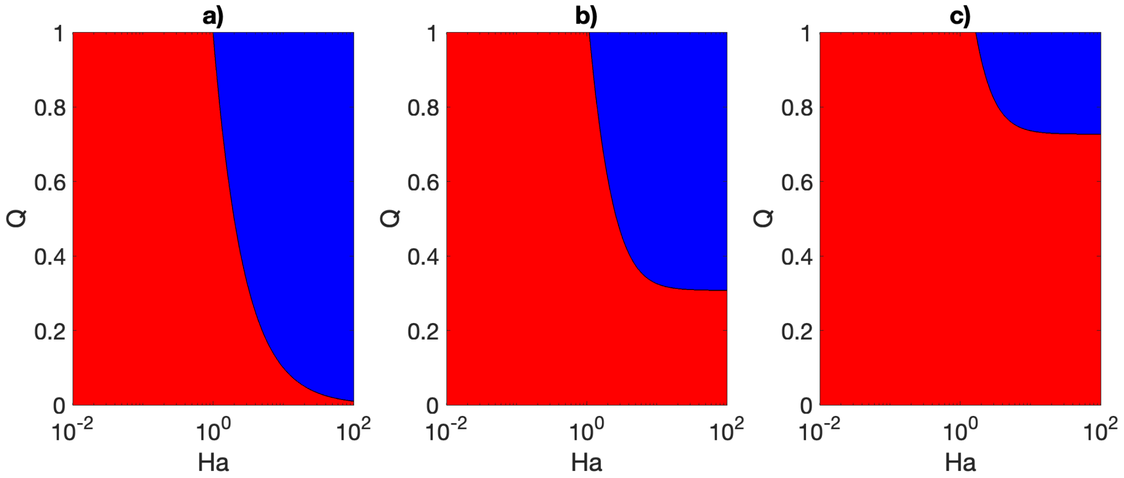

Figure S1. Solution behaviour as a function of the steady state order parameter and the Hartman number. The blue (red) regions denote parameter values with the oscillatory (stationary) solutions. The tumbling parameters used to obtain the diagrams are: a) $\xi = 0$; b) $\xi = 0.4$; c) $\xi = 0.8$.

Figure S2 shows that imposing an electric field has a similar effect to increasing the tumbling parameter, in that the field allows a steady state solution to exist at small ξ, where oscillations would otherwise occur.

1.2 Non-negligible *De*
At non-negligible Deborah numbers, there is a coupling between the order parameter and the director. This is illustrated in fig. S3. Equations (3) and (4) are nonlinear, so an analytical description of the coupled system's behaviour is not possible. Figure S3 nevertheless shows that the combined system exhibits a periodic behaviour whose dominant frequency may be extracted via FFT; the result of this is illustrated in fig. S4. Increasing the strength of the electric field (reducing Ha) reduces the frequency of the transient behaviour, ultimately leading (for $Ha \to 0$) to a fixed director angle in the steady state.

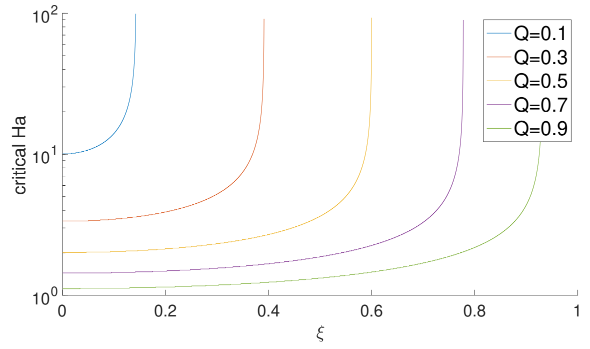

Figure S2. Critical Hartman number below which the steady state solution is obtained.

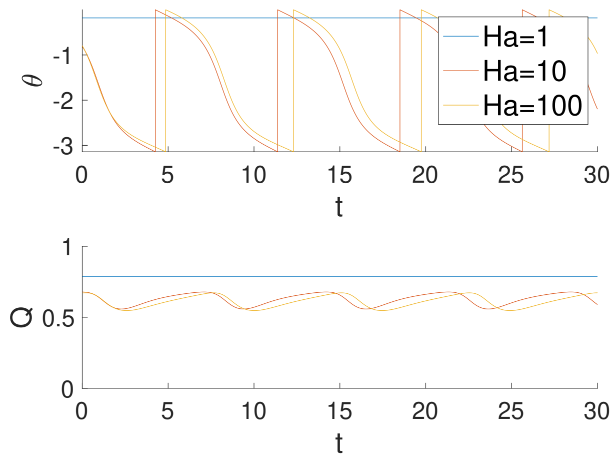

Figure S3. Evolution of a) the director angle; b) order parameter as a function of the Hartman number. $Q_{eq} = 0.621$, $\xi = 0.5$, $\dot{\gamma} = 1$, $De = 1$, $\alpha = 0$ throughout all simulations.

1.2.1 Limit $\alpha = 0$, $\xi = 0$

In the limit $\alpha = \xi = 0$, $\dot{\gamma} = 1$, the evolution equation for the director angle becomes

$$\frac{\partial \theta}{\partial t} = -\sin(2(\theta))Ha^{-1} - Q. \tag{10}$$

Solving this equation for the steady state and performing some elementary manipulation yields

$$\cos 2\theta = \sqrt{1-(QHa)^2} \tag{11}$$

Substituting for $\cos 2\theta$ into the simplified form of equation 3 provides a nonlinear equation in Q, parameterised by Ha and De. Imaginary solution components are an indicator that steady states in θ and Q are not achievable. The maximum Ha at which steady state solutions are obtained as a function of De is plotted in fig. S5, which shows that the tumbling/steady state nature only weakly depends on the Deborah number. Note that as $De \to 0$, we recover the fixed Q limit, in which the critical Ha below which oscillations cease is approximately 1.62 (for $Q_{eq} = 0.62$).

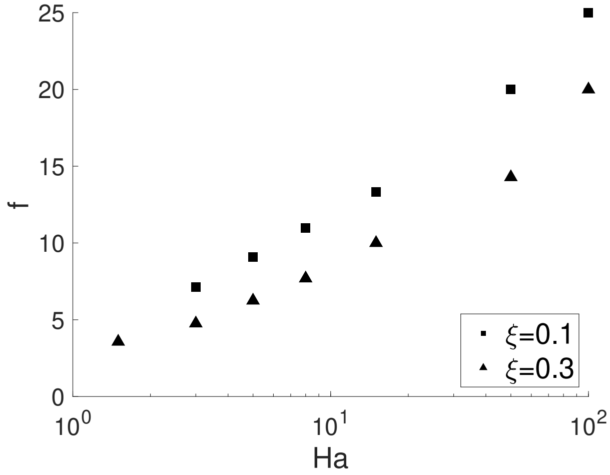

Figure S4. Oscillation frequency as a function of the Hartman number for different values of the tumbling parameter at $De = 1$.

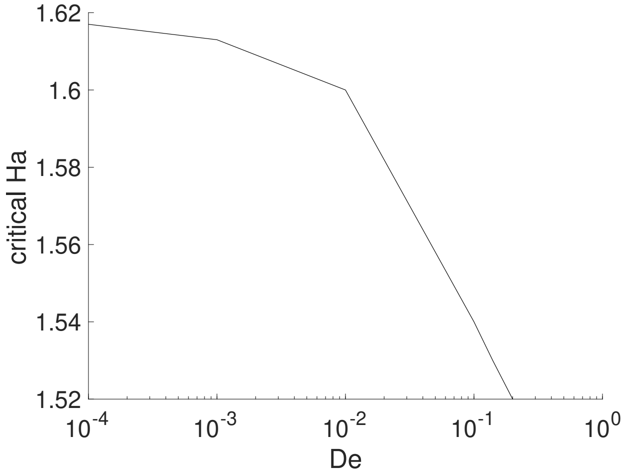

Figure S5. Critical Hartman number below which a fixed value is obtained in the steady state as a function of the Deborah number. Parameters of the nematic free energy are chosen such that $Q_{eq} = 0.62$.

References

1. Andrews, N. C., Edwards, B. J. & McHugh, A. J. Continuum dynamic behavior of homogeneous liquid-crystalline polymers under the imposition of shear and magnetic fields. *J. Rheol.* **39**, 1161–1181, DOI: 10.1122/1.550633 (1995).
2. Denniston, C., Orlandini, E. & Yeomans, J. Simulations of liquid crystals in Poiseuille flow. *Comput. Theor. Polym. Sci.* **11**, 389–395, DOI: https://doi.org/10.1016/S1089-3156(01)00004-6 (2001).
